# Correction: Study on mechanism of elemene reversing tumor multidrug resistance based on luminescence pharmacokinetics in tumor cells *in vitro* and *in vivo*

**DOI:** 10.1039/d4ra90085e

**Published:** 2024-08-12

**Authors:** Liying Chen, Zhi Chen, Shuang Zheng, Luhui Fan, Lixin Zhu, Jiandong Yu, Chaoyuan Tang, Qi Liu, Yang Xiong

**Affiliations:** a Department of Pharmaceutical Science, College of Pharmaceutical Science, Zhejiang Chinese Medical University Hangzhou 311400 Zhejiang China; b The First People’s Hospital of Jiande Jiande 311600 Zhejiang China; c Zhejiang Institute for Food and Drug Control Hangzhou 310004 Zhejiang China; d Department of Dermatology, Johns Hopkins University School of Medicine Baltimore MD 21231 USA

## Abstract

Correction for ‘Study on mechanism of elemene reversing tumor multidrug resistance based on luminescence pharmacokinetics in tumor cells *in vitro* and *in vivo*’ by Liying Chen *et al.*, *RSC Adv.*, 2020, **10**, 34928–34937, https://doi.org/10.1039/d0ra00184h.

The authors regret that due to an image selection and processing error, the panel of group B-53 min and group C-93 min were incorrect in Fig. 4. The corrected version of Fig. 4 is provided below:

**Fig. 1:**
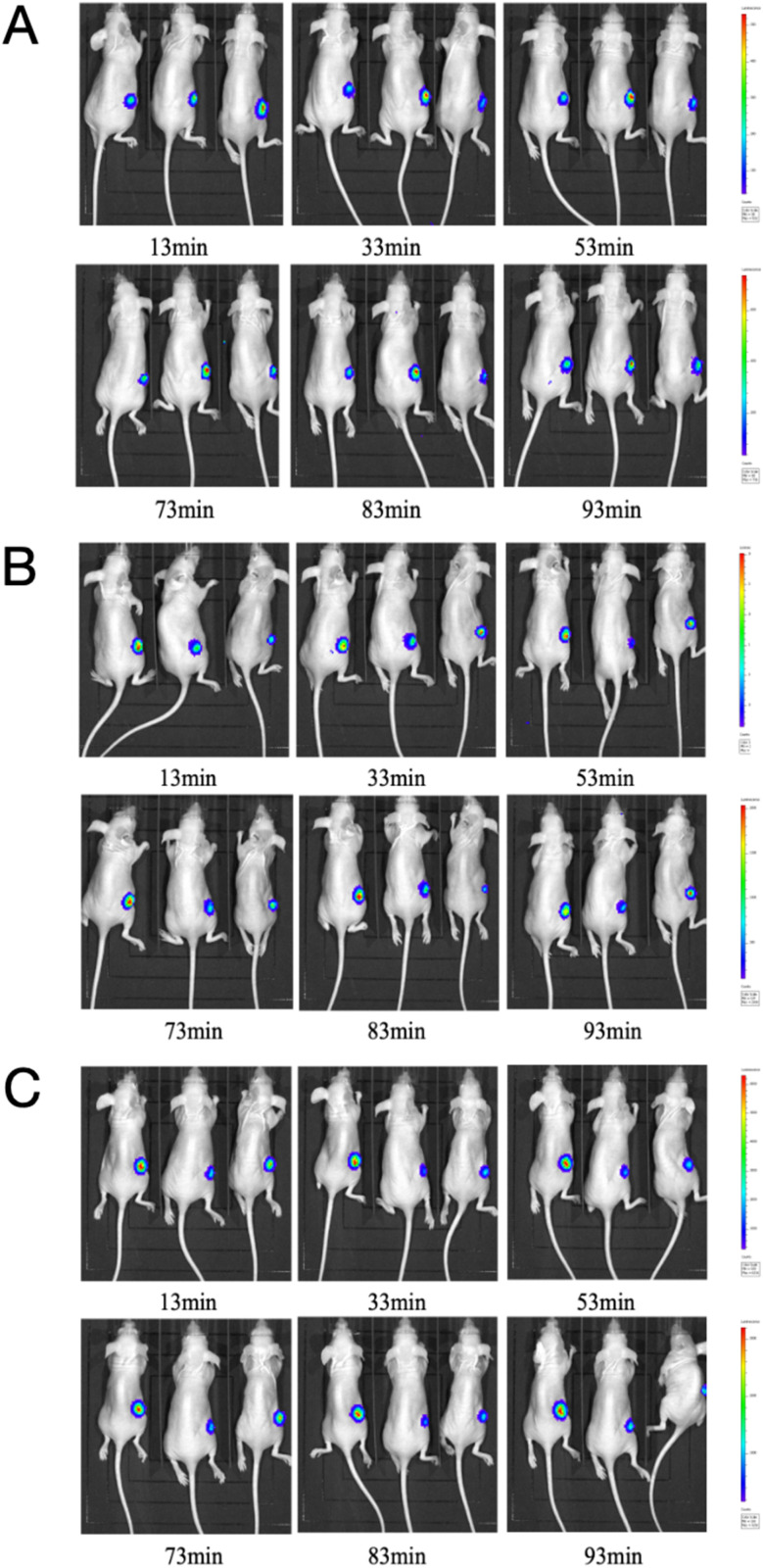
BLI of MCF-7/DOX^Fluc^ tumor-bearing nude mice at different time points within 115 min after intraperitoneal injected d-luc with a dose of 100 mg kg^−1^ (*n* = 3). (A) PBS group (i.p., QD). (B) The low ELE concentration group (ELE 10 mg kg^−1^, i.p., QD). (C) The high ELE concentration group (ELE 25 mg kg^−1^, i.p., QD).

The Royal Society of Chemistry apologises for these errors and any consequent inconvenience to authors and readers.

